# Supplementary figures and images for: Differential colitis susceptibility of Th1- and Th2-biased mice: A multi-omics approach
Source: PLoS One. 2022 Mar 9;17(3):e0264400. doi: 10.1371/journal.pone.0264400 (PMC8906622; doi:10.1371/journal.pone.0264400)

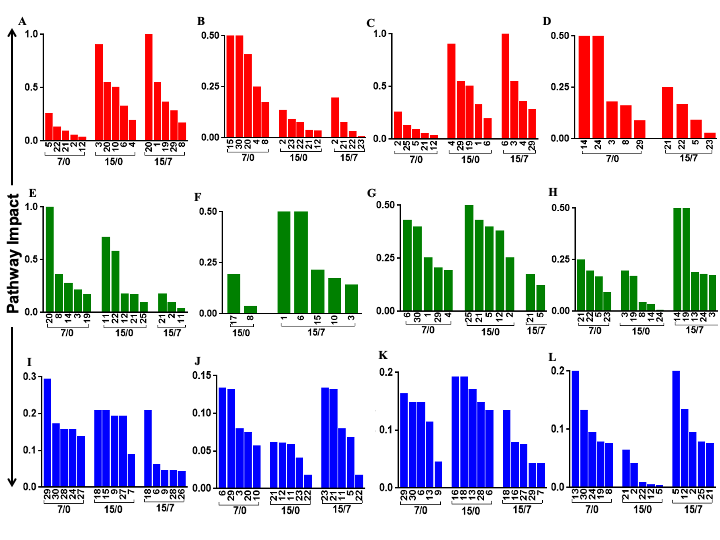

Supplement: S1 Fig — (A) Physiological changes of both mice strains were used to determine the clinical score to measure the severity of the disease progression. (B, C) Altered colon length (B) and histopathological scoring (C) further showed the disease progression in both mice strains. We presented all values as means± SEM for 3 biological replicates. We performed Two-way ANOVA followed by the Bonferroni test to determine the significance level. *corresponds to P<0.05, ** corresponds to P<0.01, *** corresponds to P<0.001. (TIFF) [file pone.0264400.s001.tiff]

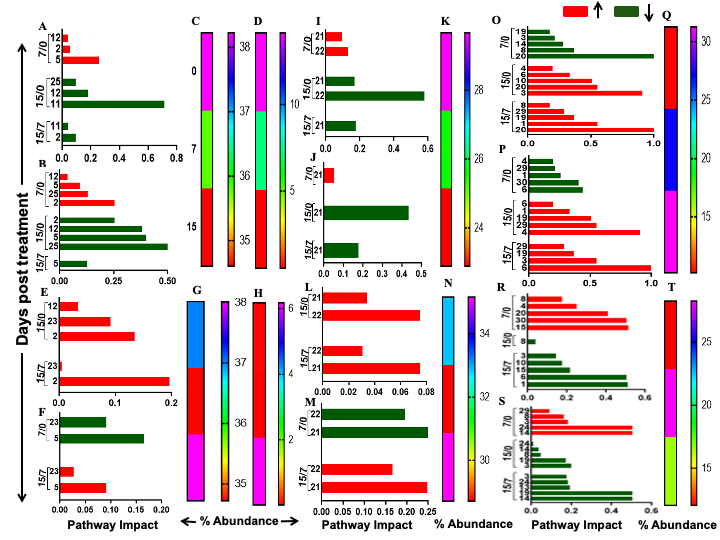

Supplement: S2 Fig — (TIFF) [file pone.0264400.s002.tiff]

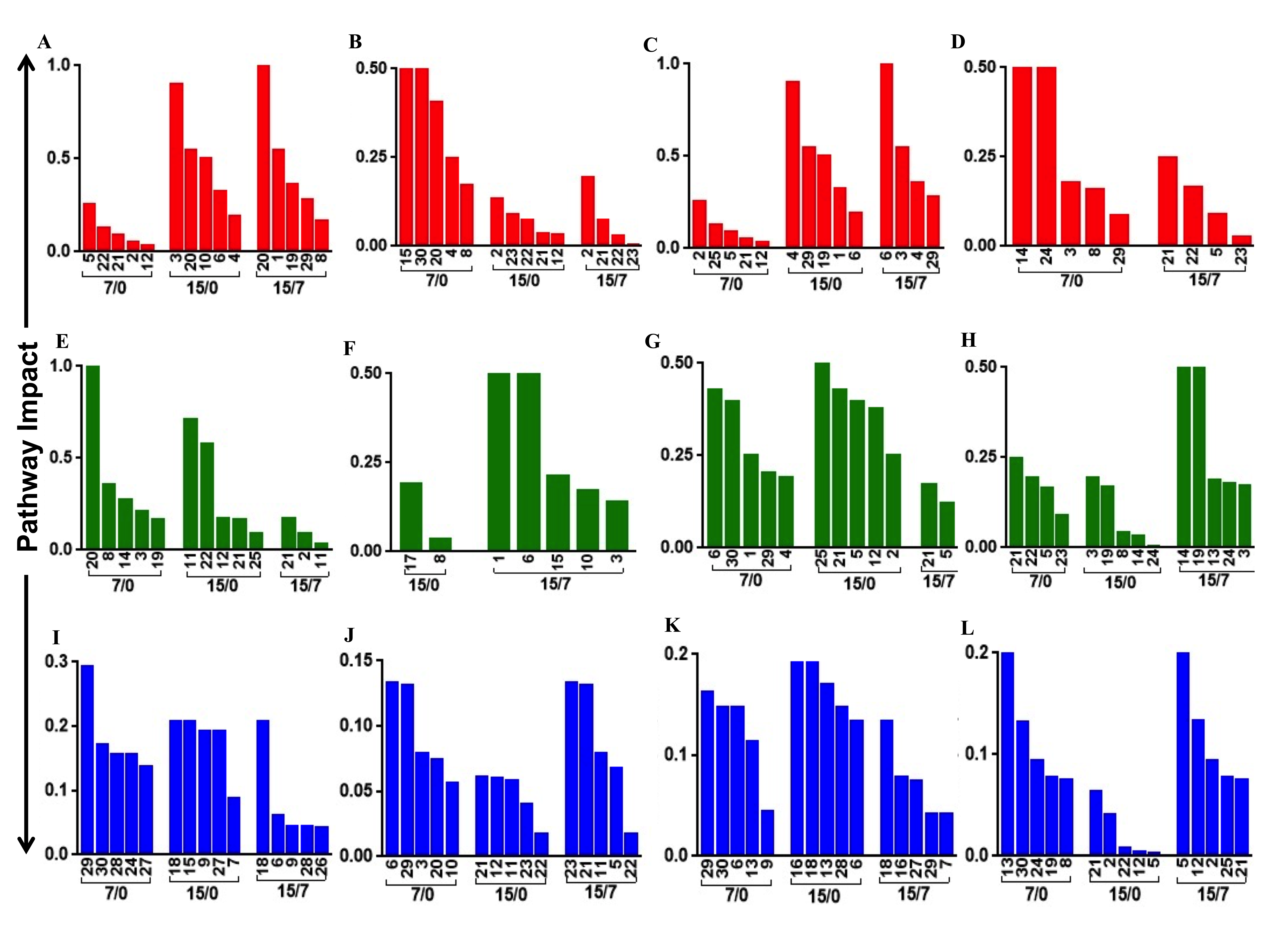

Supplement: S3 Fig — (A, B) Majorly impacted pathways due to altered metabolites’ upregulation at various treatment conditions in serum in C57BL/6 (A) and BALB/c (B) mice. (C, D) Majorly impacted pathways due to altered metabolites’ upregulation at various treatment conditions in cecal content in C57BL/6 (C) and BALB/c (D) mice. (E, F) Majorly impacted pathways due to altered metabolites’ downregulation at various treatment conditions in serum in C57BL/6 (E) and BALB/c (F) mice. (G, H) Majorly impacted pathways due to altered metabolites’ downregulation at various treatment conditions in cecal content in C57BL/6 (G) and BALB/c (H) mice. (I, J) Primarily impacted pathways due to the alteration of serum metabolites and changes in the host’s inflammatory responses in both C57BL/6 (I) and BALB/c (J) mice. (K, L) Primarily impacted pathways due to the alteration of cecal metabolites (meta-metabolites) and changes in the host’s inflammatory responses in both C57BL/6 (K) and BALB/c (L) mice. We represented the pathway names with numbers to avoid unnecessary clutters. We have enlisted the pathway names with their corresponding pathway numbers in S6 Table. (TIFF) [file pone.0264400.s003.tiff]

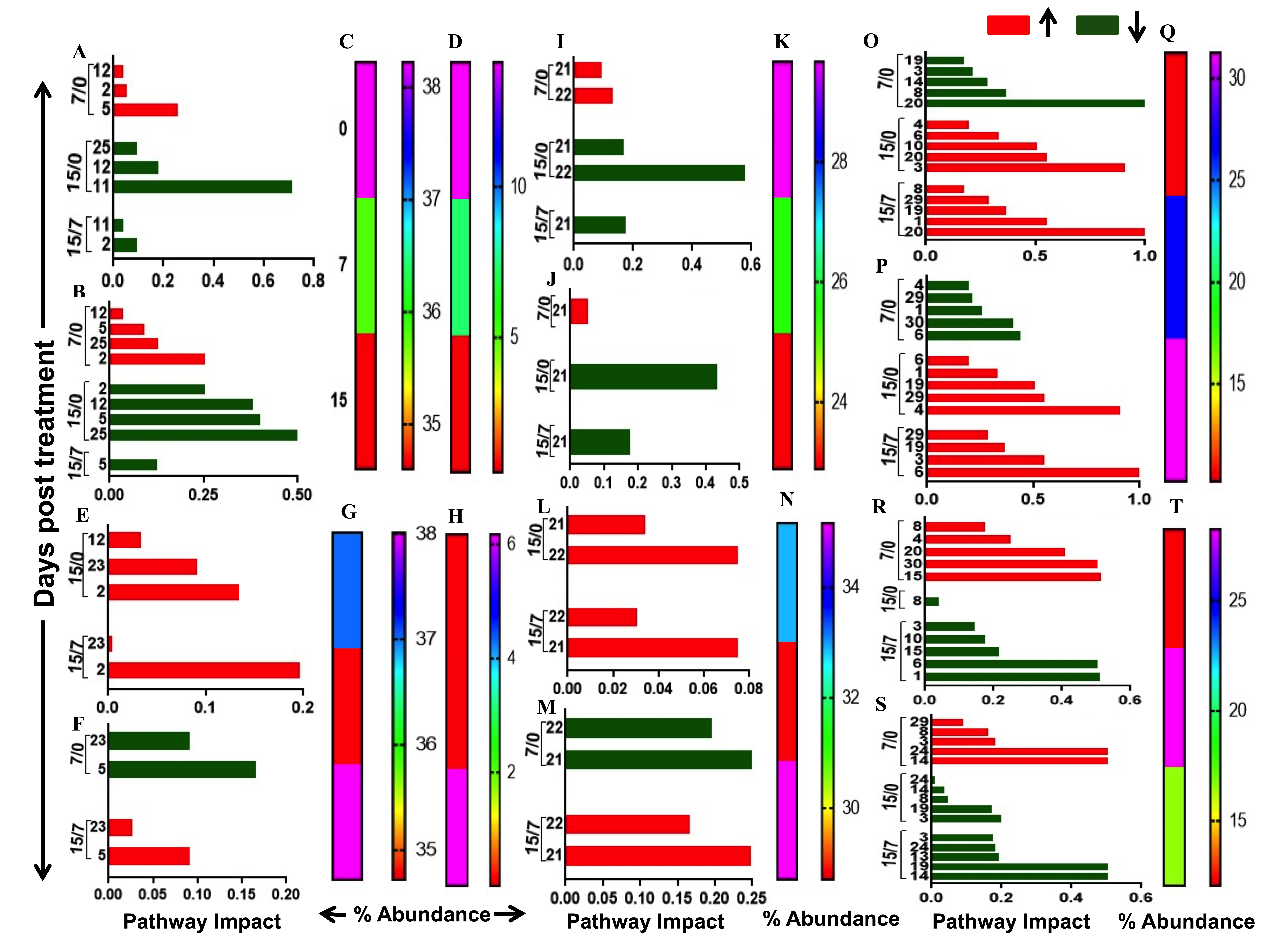

Supplement: S4 Fig — We have categorized the metabolic pathways into four different groups- i) Carbohydrate metabolism, ii) Nucleotide metabolism, iii) Amino acid metabolism, and iv) Lipid metabolism. Bacteroidetes and Verrucomicrobia are responsible for affected Carbohydrate metabolism. Firmicutes are responsible for affected Nucleotide metabolism. Proteobacteria are responsible for affected Amino acid and lipid metabolism. In C57BL/6 mice, (A, B) represents the affected carbohydrate metabolism in serum (A) and cecal (B) level, (I, J) represents the affected nucleotide metabolism in serum (I) and cecal (J) level and (O, P) represents the affected amino acid and lipid metabolism in serum (O) and cecal (P) level. (C, D, K, Q) represents the phylum level abundance of Bacteroidetes, Verrucomicrobia, Firmicutes, and Proteobacteria, respectively, in gut microbiota along with its corresponding metabolic process. In BALB/c mice, (E, F) represents the affected carbohydrate metabolism in serum (E) and cecal (F) level, (L, M) represents the affected nucleotide metabolism in serum (L) and cecal (M) level and (R, S) represents the affected amino acid and lipid metabolism in serum (R) and cecal (S) level. (G, H, N, T) represents the phylum level abundance of Bacteroidetes, Verrucomicrobia, Firmicutes, and Proteobacteria, respectively, in gut microbiota along with its corresponding metabolic process. We represented the pathway names with numbers to avoid unnecessary clutters. We have enlisted the pathway names with their corresponding pathway numbers in S6 Table. (TIFF) [file pone.0264400.s004.tiff]
