# Supplementary material for: Differential colitis susceptibility of Th1- and Th2-biased mice: A multi-omics approach
Source: PLoS One. 2022 Mar 9;17(3):e0264400. doi: 10.1371/journal.pone.0264400 (PMC8906622; doi:10.1371/journal.pone.0264400)
Supplement: S1 Table — (DOCX) [file pone.0264400.s005.docx]

**S1 Table. Sequences of forward (F) and reverse (R) primers used in gene expression studies.**

| **Genes specific for** | **NCBI_ Acc. No.** | **The sequence of the primer used (5ʹ-3ʹ)** |
| --- | --- | --- |
| **GAPDH** | **NM_008084** | **F:** CATCACTGCCACCCAGAAGACTG  **R:** ATGCCAGTGAGCTTCCCGTTCAG |
| **TNF-α** | **NM_01278601** | **F:** TGCCTATGTCTCAGCCTCTTC  **R:** GAGGCCATTTGGGAACTTCT |
| **IFN-γ** | **NM_008337.3** | **F:** CCTTTGGACCCTCTGACTTG  **R:** TTCCACATCTATGCCACTTGAG |
| **ZO-1** | **NM_009386** | **F:** GTTGGTACGGTGCCCTGAAAGA  **R:** GCTGACAGGTAGGACAGACGAT |
| **CLDN-2** | **NM_016675** | **F:** AGGACTTCCTGCTGACATCCAG  **R:** AATCCTGGCAGAACACGGTGCA |
| **IL-1β** | **NM_008361** | **F:** TGTAATGAAAGACGGCACACC  **R:** TCTTCTTTGGGTATTGCTTGG |
| **IL-6** | **NM_001314054** | **F:** CTCTGCAAGAGACTTCCATCCAGT  **R:** CGTGGTTGTCACCAGCATCA |
| **IL-12** | \| **NM_00130324** \| \| --- \| \| | **F:** CAATCACGCTACCTCCTCTTTT  **R:** CAGCAGTGCAGGAATAATGTTTC |
| **IL-17** | **NM_010552** | **F:** CAGACTACCTCAACCGTTCCAC  **R:** TCCAGCTTTCCCTCCGCATTGA |
| **IL-21** | **NM_016971** | **F:** GCTTGAGGTGTCCAACTTCCAG  **R:** ACTCCTCGGAACAGTTTCTCCC |
| **IL-10** | **NM_010548** | **F:** GCTCTTACTGACTGGCATGAG  **R:** CGCAGCTCTAGGAGCATGTG |
| **TLR-2** | **NM_011905** | **F:** GAGCATCCGAATTGCATCA  **R:** CACATGACAGAGACTCCTGAGC |
| **TLR-4** | **NM_021297** | **F:** TTCAGAACTTCAGTGGCTGGA  **R:** CTGGATAGGGTTTCCTGTCAGT |
