# Supplementary material for: Differential colitis susceptibility of Th1- and Th2-biased mice: A multi-omics approach
Source: PLoS One. 2022 Mar 9;17(3):e0264400. doi: 10.1371/journal.pone.0264400 (PMC8906622; doi:10.1371/journal.pone.0264400)
Supplement: S2 Table — (DOCX) [file pone.0264400.s006.docx]

**S2 Table. Quantitative estimation of differential responses of DSS treated C57BL/6 and BALB/c mice in terms of transcriptomics, metabolomics and meta-metabolomics using Linear Discriminant Analysis (LDA)**

| **Strain** | **Treatment Conditions** | **Distance (r) between groups on a 2-D plane** | | | | | | **The trajectory followed on the 2-D plane based on different treatment conditions (DOP- ascending order)** | | | | | |
| --- | --- | --- | --- | --- | --- | --- | --- | --- | --- | --- | --- | --- | --- |
|  |  | **T** | | **Mt** | | **M-Mt** | | **T** | | **Mt** | | **M-Mt** | |
|  |  | **i** | **ii** | **iii** | **iv** | **v** | **vi** | **i** | **ii** | **iii** | **iv** | **v** | **vi** |
| **C57BL/6** | **7 vs 0** | 1.85 | 1.95 | 2.71 | 1.68 | 1.35 | 2.74 | CW | CW | CW | ACW | ACW | CW |
|  | **15 vs 0** | 2.39 | 2.38 | 2.87 | 3.07 | 2.56 | 3.03 |  |  |  |  |  |  |
|  | **15 vs 7** | 2.14 | 2.22 | 2.29 | 3.24 | 1.54 | 3.06 |  |  |  |  |  |  |
| **BALB/c** | **7 vs 0** | 2.26 | 2.23 | 1.90 | 1.73 | 2.97 | 1.42 | ACW | ACW | CW | ACW | ACW | ACW |
|  | **15 vs 0** | 2.26 | 2.21 | 0.96 | 0.61 | 2.10 | 0.18 |  |  |  |  |  |  |
|  | **15 vs 7** | 2.31 | 2.42 | 1.46 | 1.24 | 2.43 | 1.48 |  |  |  |  |  |  |

**Abbreviations:** DOP- Days Post Treatment, T- Transcriptomics, Mt- Metabolomics, M-Mt-Meta-metabolomics, 0- Control, 7- 7 days post treatment of DSS, 15- 15 days post treatment of DSS, i- LDA was performed using all expressed genes on the particular condition, ii- LDA was performed using significantly altered genes on the particular condition, iii- LDA was performed using all metabolites present on the particular condition, iv- LDA was performed using significantly altered metabolites present on the particular condition, v- LDA was performed using all meta-metabolites present on the particular condition, vi- LDA was performed using significantly altered meta-metabolites present on the particular condition, CW- Clockwise (0-7-15 DOP), ACW- Anti-clockwise (0-15-7 DOP).
