# Supplementary material for: Differential colitis susceptibility of Th1- and Th2-biased mice: A multi-omics approach
Source: PLoS One. 2022 Mar 9;17(3):e0264400. doi: 10.1371/journal.pone.0264400 (PMC8906622; doi:10.1371/journal.pone.0264400)
Supplement: S3 Table — Significantly affected genes from transcriptomics study with function and fold change values at different treatment conditions for A. C57BL/6 and B. BALB/c mice. (DOCX) [file pone.0264400.s007.docx]

**S3A Table. Significantly affected genes from transcriptomics study with their function and fold change values at different treatment conditions for C57BL/6 mice.**

| **Sr. No.** | **Symbol** | **Full Name** | **Function** | **7d/0d** | **15d/0d** | **15d/7d** |
| --- | --- | --- | --- | --- | --- | --- |
| 1 | Mmp8 | Matrix Metallopeptidase 8 | Pro-inflammatory, stored in neutrophiles, help in the release of IL6,IL17 cytokines | 1.2 | 1.6 | 1.4 |
| 2 | Il6 | Interleukin-6 | Pro-inflammatory cytokine | 1.2 | 1.6 | 1.4 |
| 3 | Selp | Selectin P | Recruit leukocyte at the site of inflammation | 1.2 | 1.5 | 1.2 |
| 4 | Hdc | Histidine decarboxylase | Induced at an inflammatory site, especially in chronic inflammation | 1.3 | 1.5 | 1.2 |
| 5 | Cxcl5 | C-X-C motif chemokine 5 | Pro-inflammatory cytokine, activated by IL1 and TNFα | 1.5 | 1.7 | 1.1 |
| 6 | Cxcl3 | C-X-C motif chemokine 3 | Pro-inflammatory cytokine, involved in macrophage infiltration | 1.1 | 1.5 | 1.4 |
| 7 | Trpv6 | Transient Receptor Potential Cation Channel Subfamily V Member 6 | Shows pro-inflammatory response | 1.3 | 1.5 | 1.2 |
| 8 | Ngp | Neutrophilic granule protein | Increased in inflammation, works against bacterial LPS | 1 | 1.7 | 1.7 |
| 9 | Csf3 | Colony Stimulating Factor 3 | Induce expression of pro-inflammatory cytokines | 1 | 1.6 | 1.6 |
| 10 | Chil3 | Chitinase-like protein 3 | Highly expressed in inflamed tissue, especially colon lung | 1.3 | 1.6 | 1.3 |
| 11 | Mmp3 | Matrix Metallopeptidase 3 | Highly expressed in inflammatory area | 1.3 | 1.5 | 1.2 |
| 12 | S100a8 | S100 Calcium Binding Protein A8 | Highly expressed in inflammatory area, recruit leukocyte and pro-inflammatory cytokine at the site of inflammation | 1.5 | 1.9 | 1.3 |
| 13 | S100a9 | S100 Calcium Binding Protein A9 | Highly expressed in inflammatory area, recruit leukocyte and pro-inflammatory cytokine at the site of inflammation | 1.5 | 1.9 | 1.2 |
| 14 | Cxcl2 | C-X-C motif chemokine 2 | Pro-inflammatory cytokine, recruit neutrophils at the pro-inflammatory site | 1.3 | 1.7 | 1.3 |
| 15 | Igkv8-18 | Immunoglobulin kappa variable8-18 | Responsible for prolonged inflammation | 1.2 | 1.4 | 1.6 |
| 16 | Ighv15-2 | Immunoglobulin kappa variable 15-2 | Responsible for prolonged inflammation | 1.3 | 1.6 | 1.3 |
| 17 | Rnu11 | RNA, U11 Small Nuclear | Pro-inflammatory, activate TLR7 response | 1.1 | 1.7 | 1.5 |
| 18 | Scarna3a | Small Cajal Body-Specific RNA 3 | Pro-inflammatory shows oncogenic activity | 1 | 1.9 | 1.9 |
| 19 | Igkv12-44 | Immunoglobulin kappa variable 12-44 | Responsible for prolonged inflammation | 1.2 | 1.6 | 1.3 |
| 20 | Il11 | Interleukin-11 | Anti-inflammatory cytokine | -1.1 | -1.5 | 1.4 |
| 21 | Dio2 | Type II iodothyronine deiodinase | The anti-inflammatory role, suppress IL1β, COX2 expression | -1.1 | -1.6 | 1.5 |
| 22 | Retnlg | Resistin-like gamma | Involved in Th2 inflammation | -1.2 | -1.6 | 1.3 |
| 23 | Cyp2d10 | Cytochrome P450 2D10 | Anti-inflammatory | -1.1 | -1.0 | 1.0 |
| 24 | Rpl21-ps10 | Ribosomal protein L21, pseudogene 10 | Anti-inflammatory, resolve physiological inflammation | -1.5 | -1.7 | 1.1 |
| 25 | Reg3b | Regenerating islet-derived protein | Anti-inflammatory, downregulation shows more neutrophile infiltration | -1.5 | -1.7 | 1.1 |
| 26 | Itpripl2 | Inositol 1,4,5-Trisphosphate Receptor Interacting Protein Like 2 | Maintain endothelial permeability and Ca ion absorption | -1.5 | -1.1 | 1.6 |
| 27 | Muc2 | Mucin 2 | Involved in maintaining gut barrier function, Decreased at the time of gut inflammation | -1.5 | -1.1 | 1.5 |

**S3B Table. Significantly affected genes from transcriptomics study with their function and fold change values at different treatment conditions for BALB/c mice.**

| **Sr. No.** | **Symbol** | **Full Name** | **Function** | **7d/0d** | **15d/0d** | **15d/7d** |
| --- | --- | --- | --- | --- | --- | --- |
| 1 | Tpd52l1 | Tumor Protein D52 Like 1 | Pro-inflammatory in nature, related to uncontrolled cell growth | 1.1 | -1.4 | -1.5 |
| 2 | Ccl3 | C-C Motif Chemokine Ligand 3 | Pro-inflammatory cytokine, recruit leukocytes at the pro-inflammatory site | 1.6 | -1.6 | -1.0 |
| 3 | Tshr | TSH receptor | Involved in Th2inflammation | -1.6 | 2.0 | -1.2 |
| 4 | Padi4 | Peptidyl Arginine Deiminase 4 | Pro-inflammatory, involved in granulocyte and macrophage development | 1.2 | -1.4 | -1.6 |
| 5 | Fmo4 | Flavin Containing Dimethylaniline Monoxygenase 4 | Involved in TLR4 dependent inflammatory response, high TLR4 downregulate the expression of this gene | 1.3 | -1.4 | -1.7 |
| 6 | Hc | Hemolytic complement | Pro-inflammatory activates IL1β response, recruit neutrophils at the inflammatory site | 1.2 | -1.5 | -1.7 |
| 7 | Ntsr1 | Neurotensin receptor type 1 | Pro-inflammatory, highly expressed in inflamed colon | 1.1 | -1.4 | -1.5 |
| 8 | Adam18 | Disintegrin and metalloproteinase domain-containing protein 18 | Pro-inflammatory, help in the release of TNFα | 1.9 | -1.5 | -2.8 |
| 9 | Ninj2 | Ninjurin 2 | Pro-inflammatory, regulate expression of IL6, IL1β, TNFα | 1.5 | -1.3 | -2.0 |
| 10 | Try4 | Trypsin 4 | Pro-inflammatory, induce the inflammation process | 1.1 | -2.2 | -2.5 |
| 11 | Hoxb8 | Homeobox protein Hox-B8 | Pro-inflammatory, highly expressed in inflammatory colon | 1.9 | -1.2 | -2.1 |
| 12 | Hamp2 | Hepcidin antimicrobial peptide 2 | Increased in inflammation, works against bacterial LPS | 1.4 | -1.4 | -1.9 |
| 13 | Lep | Leptin | Pro-inflammatory, Induce IL2,IL12,IFNγ cytokine production | 1.6 | -1.9 | -1.2 |
| 14 | Paqr9 | Progestin And AdipoQ Receptor Family Member 9 | Pro-inflammatory | 1.2 | -1.5 | -1.3 |
| 15 | Klra3 | killer cell lectin-like receptor | Pro-inflammatory, involve in NK cell activation | 1.1 | -1.6 | -1.4 |
| 16 | Igkv9-123 | Immunoglobulin kappa variable 9-123 | Responsible for prolonged inflammation | 1.1 | -1.8 | -1.7 |
| 17 | Mcpt-ps1 | Mast cell protease, pseudogene 1 | Anti-inflammatory | -1.0 | 1.5 | -1.5 |
| 18 | Psg17 | Pregnancy specific glycoprotein 17 | Anti-inflammatory, control infections and inflammatory conditions | -1.8 | 1.0 | -1.8 |
| 19 | 2310034C09Rik | - | Anti-inflammatory, downregulation cause inflammation | -2.5 | 1.2 | -2.9 |
| 20 | Rn7sk | RNA Component Of 7SK Nuclear Ribonucleoprotein | Anti-inflammatory | -1.4 | 1.6 | -1.1 |
| 21 | Krt90 | Keratin 90 | Anti-inflammatory, downregulation cause inflammation | -1.5 | 1.2 | -1.8 |
| 22 | Sptssb | Serine Palmitoyl transferase Small Subunit B | Anti-inflammatory, suppression of it associated with leaky gut and suppression of MUC2 | -1.3 | 1.4 | -1.9 |
| 23 | Tmprss13 | Transmembrane Serine Protease 13 | Anti-inflammatory, promote cell survival, restrict apoptosis | -2.3 | 1.6 | -3.6 |
| 24 | Agr3 | Anterior Gradient 3, Protein Disulphide Isomerase Family Member | Anti-inflammatory | -1.1 | 1.5 | -1.6 |
| 25 | Ceacam12 | Carcinoembryonic antigen-related cell adhesion molecule 12 | Anti-inflammatory | -1.4 | 1.7 | -2.4 |
| 26 | Tpsab1 | Tryptase Alpha/Beta 1 | Anti-inflammatory, control infections and sepsis | -1.6 | 1.2 | -1.9 |
| 27 | Slc47a1 | Solute Carrier Family 47 Member 1 | Related to anti-inflammation | -1.4 | 1.5 | -2.1 |
| 28 | Muc6 | Mucin 6, oligomeric mucus/gel-forming | Involved in maintaining gut barrier function, Decreased at the time of gut inflammation | -1.1 | 1.3 | -1.5 |
| 29 | Scnn1g | Sodium Channel Epithelial 1 Subunit Gamma | Associated to leaky gut. Maintain electrolyte (Na, K) balance in the gut | -1.3 | 1.2 | -1.6 |
