# Supplementary material for: Differential colitis susceptibility of Th1- and Th2-biased mice: A multi-omics approach
Source: PLoS One. 2022 Mar 9;17(3):e0264400. doi: 10.1371/journal.pone.0264400 (PMC8906622; doi:10.1371/journal.pone.0264400)
Supplement: S4 Table — Significantly affected serum metabolites from metabonomics study with their function and fold change values at different treatment conditions for A. C57BL/6 and B. BALB/c mice. (DOCX) [file pone.0264400.s008.docx]

**S4A Table. Significantly affected serum metabolites from metabonomics study with their function and fold change values at different treatment conditions for C57BL/6 mice.**

| **Sr. No.** | **Metabolites** | **Function** | **7d/0d** | **15d/0d** | **15d/7d** |
| --- | --- | --- | --- | --- | --- |
| 1 | Fumarate | Anti-inflammatory, alleviates colitis by activating anti-oxidant and anti-inflammatory pathways | 3.3 | - | 2.0 |
| 2 | Creatine phosphate | Anti-inflammatory, alleviates the severity of colitis | 3.3 | - | 3.8 |
| 3 | Indole-3-lactate | Anti-inflammatory, controls autophagy | 1.9 | 1.7 | 4.4 |
| 4 | Lysine | Anti-inflammatory, controls gut mucosal inflammation | -2.7 | - | - |
| 5 | Glutamine | Anti-inflammatory, help in mucosal healing in colitis | 1.8 | - | - |
| 6 | 2,6-Dihydroxybenzoate | Anti-inflammatory, provide protection against colitis | 3.0 | 1.7 | 1.8 |
| 7 | 2,3,4-Trihydroxybenzoate | Anti-inflammatory, provide protection against colitis | 2.4 | -1.7 | 4.0 |
| 8 | Pyrocatechol | Anti-inflammatory, activate ROS to control intestinal inflammation | 1.6 | -1.9 | 3.1 |
| 9 | Adenine | anti-inflammatory, alleviates inflammation | 3.3 | - | -1.9 |
| 10 | Inosine | Anti-inflammatory, ameliorate inflammation | 2.2 | 1.8 |  |
| 11 | Protocatechuate | Anti-inflammatory, controls CRP, IL6,TNFa level | 2.4 | - | 1.7 |
| 12 | Imidazole | Anti-inflammatory, prevents inflammosome formation in colitis | -2.2 | - | -1.9 |
| 13 | Hypoxanthine | Improves gut-barrier function | 2.3 | - | 2.1 |
| 14 | 2'-Deoxyuridine | Cause neurogastrointestinal encephalopathy in colitis patients | -2.8 | -1.5 | - |
| 15 | Niacinamide | Increase colitis related inflammation and angiogenesis | 5.4 | - | 6.0 |
| 16 | Tyrosine | Increased in colitis patient, good marker for diagnosis | 1.7 | -1.6 | 1.7 |
| 17 | 3-Indoxylsulfate | Indication of gut dysbiosis and increased amount of pathogenic bacteria in gut | 2.2 | -3.4 | 7.5 |
| 18 | Uracil | Induce inflammation in colon | 2.5 | - | 1.7 |
| 19 | Cytosine | Induce inflammation in colon and peritoneum | 3.4 | 3.3 | - |
| 20 | NADP+ | Pro-inflammatory, activate localized inflammation process | 1.8 | - | - |
| 21 | Quinolinate | Pro-inflammatory, elevated at the time of infection and inflammation | 4.6 | -1.7 | 7.6 |
| 22 | Nicotinate | Related to colitis disease severity, inflammation | 6.5 | 3.8 | 1.7 |
| 23 | 6-Hydroxynicotinate | Related to colitis disease severity, inflammation | 3.4 | 1.8 | 1.9 |
| 24 | Nicotinamide N-oxide | Related to colitis disease severity, positively correlated with hypoxia, leukocyte infiltration and inflammation | 5.5 | 2.4 | 2.3 |
| 25 | 1-Methylnicotinamide | Related to colitis disease severity,positively correlated with hypoxia, leukocyte infiltration and inflammation | 2.7 | - | 1.8 |
| 26 | Serine | anti-inflammatory, alleviates oxidative stress and inflammatory response | - | -1.5 | - |
| 27 | Agmatine | Anti-inflammatory, reduce intestinal inflammation | - | -3.8 | 4.7 |
| 28 | Catechol | Anti-inflammatory, activate ROS to control intestinal inflammation | - | -1.6 | 2.0 |
| 29 | Maleate | Anti-inflammatory, ameliorate inflammation | - | 2.6 | -1.9 |
| 30 | Epicatechin | Anti-inflammatory, ameliorate inflammation by blocking NF-kB pathway | - | 2.2 | -1.8 |
| 31 | Arabinose | Exert anti-inflammatory effect in colitis | - | -1.8 | - |
| 32 | Tryptophan | Reduce inflammation in colitis and help in maintaining gut barrier function | - | -3.3 | 2.6 |
| 33 | Phenylalanine | Cause inflammation in intestinal mucosa | - | -3.2 | 2.8 |
| 34 | Ascorbate | Colitis patient content more ascorbate in the intestine | - | -1.5 |  |
| 35 | Glucose | High glucose exacerbate inflammation by activating TGF-B pathway | - | -2.0 | - |
| 36 | 5-Hydroxytryptophan | Increase the severity of colitis | - | -1.8 | 2.4 |
| 37 | Xylose | Increased xylose indicates malabsorption of intestine due to inflammation. | - | -1.6 | 1.7 |
| 38 | 3-Hydroxykynurenine | Pro-inflammatory, elevated in colitis | - | -1.7 | - |
| 39 | Pyroglutamate | Pro-inflammatory, indicator of cellular inflammatory responses | - | -6.2 | 4.0 |
| 40 | Homocysteine | Pro-inflammatory, participate in mucosal inflammation in colitis | - | -1.6 | 1.8 |

**S4B Table. Significantly affected serum metabolites from metabonomics study with their function and fold change values at different treatment conditions for BALB/c mice.**

| **Sr. No.** | **Metabolites** | **Function** | **7d/0d** | **15d/0d** | **15d/7d** |
| --- | --- | --- | --- | --- | --- |
| 1 | Fumarate | Anti-inflammatory, alleviates colitis by activating anti-oxidant and anti-inflammatory pathways | 3.3 | 5.1 | 1.6 |
| 2 | Trigonelline | Anti-inflammatory, attenuates the inflammatory effect of Tnf-α, Il/1β and, Tlr4 | 2.0 | -5.0 | -10.1 |
| 3 | Indole-3-lactate | Anti-inflammatory, controls autophagy | 1.6 | - | - |
| 4 | Aspartate | Anti-inflammatory, down-regulated TLR 4, NOD1, and MyD88 expression | 2.0 | - | -2.4 |
| 5 | 2,3,4-Trihydroxybenzoate | Anti-inflammatory, provide protection against colitis | 2.0 | 11.3 | 5.5 |
| 6 | Catechol | Anti-inflammatory, activate ROS to control intestinal inflammation | 1.7 | 13.1 | 7.7 |
| 7 | Adenine | anti-inflammatory, alleviates inflammation | 2.0 | 4.2 | 2.2 |
| 8 | Maleate | Anti-inflammatory, ameliorate inflammation | 3.3 | 6.7 | 2.0 |
| 9 | Protocatechuate | Anti-inflammatory, controls CRP, IL6,TNFa level | 3.4 | 9.2 | 2.7 |
| 10 | Glutamate | Anti-inflammatory, reduce colitis disease score by activating anti-oxidants and cell proliferation | 2.8 | - | -1.9 |
| 11 | N-Acetylglucosamine | Anti-inflammatory, used as a treatment for IBD | 5.1 | - | -7.2 |
| 12 | Hypoxanthine | Improves gut-barrier function | 2.4 | 4.3 | 1.8 |
| 13 | Ascorbate | Colitis patient content more ascorbate in the intestine | 3.6 | - | -4.5 |
| 14 | Niacinamide | Increase colitis related inflammation and angiogenesis | 2.4 | 10.3 | 4.2 |
| 15 | 5-Hydroxytryptophan | Increase the severity of colitis | 1.5 | 1.7 | - |
| 16 | Tyrosine | Increased in colitis patient, good marker for diagnosis | 1.9 | - | - |
| 17 | Uracil | Induce inflammation in colon | 2.7 | 4.3 | 1.6 |
| 18 | Cytosine | Induce inflammation in colon and peritoneum | 5.1 | 6.6 | - |
| 19 | Quinolinate | Pro-inflammatory, elevated at the time of infection and inflammation | 2.5 | 2.1 | - |
| 20 | N-Acetylaspartate | Pro-inflammatory, related to tumor growth and adipogenesis | 5.8 | - | -8.5 |
| 21 | Homocysteine | Pro-inflammatory, participate in mucosal inflammation in colitis | 1.8 | -1.7 | -3.1 |
| 22 | 6-Hydroxynicotinate | Related to colitis disease severity, inflammation | 11.4 | - | -2.9 |
| 23 | Nicotinate | Related to colitis disease severity, inflammation | 5.0 | 5.9 | - |
| 24 | Nicotinamide N-oxide | Related to colitis disease severity, positively correlated with hypoxia, leukocyte infiltration and inflammation | 4.1 | 2.1 | -1.9 |
| 25 | 2,6-Dihydroxybenzoate | Anti-inflammatory, provide protection against colitis | - | 10.7 | 6.5 |
| 26 | Gallate | Anti-inflammatory, reduced the expression of COX2, IL-6, TNFα | - | 7.9 | 6.0 |
| 27 | Pyrocatechol | Anti-inflammatory, activate ROS to control intestinal inflammation | - | 12.0 | 9.6 |
| 28 | Adenine | anti-inflammatory, alleviates inflammation | - | 4.2 | 2.2 |
| 29 | 3-Indoxylsulfate | Indication of gut dysbiosis and increased amount of pathogenic bacteria in gut | - | 7.7 | 5.1 |
| 30 | Epicatechin | Anti-inflammatory, protective effect mediated by increasing antioxidation and by the inhibition of NF-κB pathway | - | - | 2.8 |
| 31 | Arabinose | Exert anti-inflammatory effect in colitis | - | - | -1.8 |
