# Supplementary material for: Differential colitis susceptibility of Th1- and Th2-biased mice: A multi-omics approach
Source: PLoS One. 2022 Mar 9;17(3):e0264400. doi: 10.1371/journal.pone.0264400 (PMC8906622; doi:10.1371/journal.pone.0264400)
Supplement: S5 Table — Significantly affected cecal metabolites from metabonomics study with their function and fold change values at different treatment conditions for A. C57BL/6 and B. BALB/c mice. (DOCX) [file pone.0264400.s009.docx]

**S5A Table. Significantly affected cecal metabolites from metabonomics study with their function and fold change values at different treatment conditions for C57BL/6 mice.**

| **Sr. No.** | **Metabolites** | **Function** | **7d/0d** | **15d/0d** | **15d/7d** |
| --- | --- | --- | --- | --- | --- |
| 1 | Guanosine | Anti-inflammatory and anti-oxidative, block the activation of NF-KB pathway | 28.3 | - | - |
| 2 | Serine | anti-inflammatory, alleviates oxidative stress and inflammatory response | -3.8 | -2.5 | - |
| 3 | Indole-3-acetate | Anti-inflammatory, reduce macrophage inflammation | -3.4 | - | -34.2 |
| 4 | Vanillate | Anti-inflammatory, stops neutrophile migration | -2.1 | 21.4 | -45.5 |
| 5 | Lactulose | Anti-inflammatory, used as prebiotic for colitis treatment | -3.4 | -7.0 | -2.0 |
| 6 | Adenine | anti-inflammatory, alleviates inflammation | 75.9 | - | - |
| 7 | Inosine | Anti-inflammatory, ameliorate inflammation | 21.0 | - | - |
| 8 | Imidazole | Anti-inflammatory, prevents inflammosome formation in colitis | 2.9 | - | 14.2 |
| 9 | Arabinose | Exert anti-inflammatory effect in colitis | -3.0 | -12.1 | 4.1 |
| 10 | Hypoxanthine | Improves gut-barrier function | 78.5 |  |  |
| 11 | Niacinamide | Increase colitis related inflammation and angiogenesis | 26.4 | 15.4 | 4.2 |
| 12 | 3-Indoxylsulfate | Indication of gut dysbiosis and increased amount of pathogenic bacteria in gut | 2.3 | 54.7 | 23.6 |
| 13 | Uracil | Induce inflammation in colon | 2.0 | 44.0 | 90.0 |
| 14 | Cytidine | Induce inflammation in colon and peritoneum | 11.8 | 8.4 | - |
| 15 | Cytosine | Induce inflammation in colon and peritoneum | 1.9 | 70.8 | 38.1 |
| 16 | Quinolinate | Pro-inflammatory, elevated at the time of infection and inflammation | 55.8 | 25.5 | - |
| 17 | 3-Hydroxykynurenine | Pro-inflammatory, elevated in colitis | -3.5 | 29.0 | 101.4 |
| 18 | Mannitol | Pro-inflammatory, elevated in colitis | -3.5 | -3.2 | - |
| 19 | Nicotinate | Related to colitis disease severity, inflammation | 60.3 | 41.3 | 4.3 |
| 20 | 6-Hydroxynicotinate | Related to colitis disease severity, inflammation | 47.7 | 11.8 | 11.8 |
| 21 | Nicotinamide N-oxide | Related to colitis disease severity, positively correlated with hypoxia, leukocyte infiltration and inflammation | 32.6 | 11.9 | 13.6 |
| 22 | Ascorbate | Colitis patient content more ascorbate in the intestine | -3.5 | -3.6 | - |
| 23 | Taurine | Anti-inflammatory, attenuates carcinogenicity in colitis | - | -32.7 | -22.0 |
| 24 | Glycine | Anti-inflammatory, attenuates the activation of pro-inflammatory cytokine and chemokine in colitis | - | -8.6 | -2.5 |
| 25 | Cellobiose | Anti-inflammatory, attenuates the activation of pro-inflammatory cytokines in colitis | - | -41.1 | -28.6 |
| 26 | Trehalose | Anti-inflammatory, maintain autophagic flux and reduce the severity of colitis | - | -24.5 | -13.6 |
| 27 | myo-Inositol | Anti-inflammatory, reduces β-catenin activation in colitis | - | -27.3 |  |
| 28 | Gallate | Anti-inflammatory, reduced the expression of COX2, IL-6, TNFα | - | 21.4 | -18.0 |
| 29 | Pyrocatechol | Anti-inflammatory, activate ROS to control intestinal inflammation | - | 228.9 | -212.2 |
| 30 | Catechol | Anti-inflammatory, activate ROS to control intestinal inflammation | - | 227.3 | -211.5 |
| 31 | Histamine | Cause allergic enteropathy in colitis | - | -28.7 | -13.2 |
| 32 | Glucose | High glucose exacerbate inflammation by activating TGF-B pathway | - | -37.2 | -40.6 |
| 33 | Xylose | Increased xylose indicates malabsorption of intestine due to inflammation. | - | -42.5 | -48.3 |
| 34 | Quinolinate | Pro-inflammatory, elevated at the time of infection and inflammation | - | 25.5 | - |
| 35 | Homocysteine | Pro-inflammatory, participate in mucosal inflammation in colitis | - | 3.1 | 13.1 |
| 36 | N-Acetylaspartate | Pro-inflammatory, related to tumor growth and adipogenesis | - | 9.5 | 8.5 |
| 37 | Pyroglutamate | Pro-inflammatory, indicator of cellular inflammatory responses | - | 4.0 | 6.0 |
| 38 | Tyrosine | Increased in colitis patient, good marker for diagnosis | - | 1.9 | 3.9 |
| 39 | myo-Inositol | Anti-inflammatory, reduces β-catenin activation in colitis | - | - | -24.8 |
| 40 | Maleate | Anti-inflammatory, ameliorate inflammation | - | - | -5.4 |
| 41 | Protocatechuate | Anti-inflammatory, controls CRP, IL6,TNFa level | - | - | 63.0 |

**S5B Table. Significantly affected cecal metabolites from metabonomics study with their function and fold change values at different treatment conditions for BALB/c mice.**

| **Sr. No.** | **Metabolites** | **Function** | **7d/0d** | **15d/0d** | **15d/7d** |
| --- | --- | --- | --- | --- | --- |
| 1 | Guanosine | Anti-inflammatory and anti-oxidative, block the activation of NF-KB pathway | -4.0 | -6.0 | - |
| 2 | Fumarate | Anti-inflammatory, alleviates colitis by activating anti-oxidant and anti-inflammatory pathways | -5.3 | - | 6.2 |
| 3 | Serine | anti-inflammatory, alleviates oxidative stress and inflammatory response | 4.0 | -5.6 | -22.5 |
| 4 | Lactulose | Anti-inflammatory, used as prebiotic for colitis treatment | 13.0 | - | -35.7 |
| 5 | Pyrocatechol | Anti-inflammatory, activate ROS to control intestinal inflammation | -5.6 | - | 6.2 |
| 6 | Catechol | Anti-inflammatory, activate ROS to control intestinal inflammation | -5.6 | - | 6.2 |
| 7 | Adenine | anti-inflammatory, alleviates inflammation | -6.4 | - | 5.3 |
| 8 | Protocatechuate | Anti-inflammatory, controls CRP, IL6,TNFa level | -6.2 | - | 6.4 |
| 9 | Imidazole | Anti-inflammatory, prevents inflammosome formation in colitis | -6.0 | - | 6.4 |
| 10 | Ascorbate | Colitis patient content more ascorbate in the intestine | 5.7 | -12.5 | -74.0 |
| 11 | Arabinose | Exert anti-inflammatory effect in colitis | 20.0 | - | -57.9 |
| 12 | Hypoxanthine | Improves gut-barrier function | -6.3 | - | - |
| 13 | 3-Indoxylsulfate | Indication of gut dysbiosis and increased amount of pathogenic bacteria in gut | -6.2 | - | 6.5 |
| 14 | Uracil | Induce inflammation in colon | -6.4 | - | 6.4 |
| 15 | Cytosine | Induce inflammation in colon and peritoneum | -6.2 | - | 6.3 |
| 16 | Mannitol | Mannitol excretion is high in colitis | 6.2 | - | -54.2 |
| 17 | Quinolinate | Pro-inflammatory, elevated at the time of infection and inflammation | -6.5 | - | 5.1 |
| 18 | 3-Hydroxykynurenine | Pro-inflammatory, elevated in colitis | 6.8 | - | -50.2 |
| 19 | 6-Hydroxynicotinate | Related to colitis disease severity, inflammation | -4.3 | - | 3.9 |
| 20 | Nicotinamide N-oxide | Related to colitis disease severity, positively correlated with hypoxia, leukocyte infiltration and inflammation | -7.3 | -1.5 | 4.3 |
| 21 | Homocysteine | Pro-inflammatory, participate in mucosal inflammation in colitis | 3.1 | - | - |
| 22 | N-Acetylaspartate | Pro-inflammatory, related to tumor growth and adipogenesis | 45.5 | - | - |
| 23 | Niacinamide | Increase colitis related inflammation and angiogenesis | 84.2 | -4.1 | - |
| 24 | Tyrosine | Increased in colitis patient, good marker for diagnosis | 3.9 | - | - |
| 25 | Pyroglutamate | Pro-inflammatory, indicator of cellular inflammatory responses | 10.0 | - | - |
| 26 | Inosine | Anti-inflammatory, ameliorate inflammation | - | -5.0 | - |
| 27 | Cytidine | Induce inflammation in colon and peritoneum | - | -7.7 | -7.9 |
| 28 | Nicotinate | Related to colitis disease severity, inflammation | - | -4.2 | -3.6 |
| 29 | 2,6-Dihydroxybenzoate | Anti-inflammatory, provide protection against colitis | - | - | 6.4 |
| 30 | 2,3,4-Trihydroxybenzoate | Anti-inflammatory, provide protection against colitis | - | - | 6.4 |
| 31 | Gallate | Anti-inflammatory, reduced the expression of COX2, IL-6, TNFα | - | - | 6.2 |
| 32 | Vanillate | Anti-inflammatory, stops neutrophile migration | - | - | -14.4 |
| 33 | Maleate | Anti-inflammatory, ameliorate inflammation | - | - | 4.5 |
