# Supplementary material for: Differential colitis susceptibility of Th1- and Th2-biased mice: A multi-omics approach
Source: PLoS One. 2022 Mar 9;17(3):e0264400. doi: 10.1371/journal.pone.0264400 (PMC8906622; doi:10.1371/journal.pone.0264400)
Supplement: S6 Table — (DOCX) [file pone.0264400.s010.docx]

**S6 Table. Pathway name and its corresponding pathway no. used in the main figure panel.**

| **Pathway No.** | **Pathway Name** |
| --- | --- |
| 1 | Alanine, aspartate and glutamate metabolism |
| 2 | Amino sugar and nucleotide sugar metabolism |
| 3 | Arginine and proline metabolism |
| 4 | Arginine metabolism |
| 5 | Ascorbate and aldarate metabolism |
| 6 | beta-Alanine metabolism |
| 7 | Chemokine signalling pathway |
| 8 | Cysteine and methionine metabolism |
| 9 | Cytokine-cytokine receptor interaction |
| 10 | D-Glutamine and D-glutamate metabolism |
| 11 | Fructose and mannose metabolism |
| 12 | Galactose metabolism |
| 13 | Glycerolipid metabolism |
| 14 | Glycine, serine and threonine metabolism |
| 15 | Histidine metabolism |
| 16 | IL-17 signalling pathway |
| 17 | Inositol phosphate metabolism |
| 18 | NOD-like receptor signalling pathway |
| 19 | Phenylalanine metabolism |
| 20 | Phenylalanine, tyrosine and tryptophan metabolism |
| 21 | Purine metabolism |
| 22 | Pyrimidine metabolism |
| 23 | Pyruvate metabolism |
| 24 | Sphingolipid metabolism |
| 25 | Starch and sucrose metabolism |
| 26 | Th17 cell differentiation |
| 27 | TNF signalling pathway |
| 28 | Toll-like receptor signalling pathway |
| 29 | Tryptophan metabolism |
| 30 | Tyrosine metabolism |
