# Supplementary material for: Differential colitis susceptibility of Th1- and Th2-biased mice: A multi-omics approach
Source: PLoS One. 2022 Mar 9;17(3):e0264400. doi: 10.1371/journal.pone.0264400 (PMC8906622; doi:10.1371/journal.pone.0264400)
Supplement: S7 Table — (DOCX) [file pone.0264400.s011.docx]

**S7 Table. Disease severity score based on stool texture and rectal bleeding**

| **Condition** | **Score** |
| --- | --- |
| Normal + no hemoccult | 0 |
| Soft + no hemoccult | 1 |
| Soft + hemoccult | 2 |
| Soft+ very little amount of rectal bleeding | 3 |
| Very soft+ blood | 4 |
| Watery+ rectal bleeding | 5 |
