# Supplementary material for: Differential colitis susceptibility of Th1- and Th2-biased mice: A multi-omics approach
Source: PLoS One. 2022 Mar 9;17(3):e0264400. doi: 10.1371/journal.pone.0264400 (PMC8906622; doi:10.1371/journal.pone.0264400)
Supplement: S8 Table — Correlation analysis between inflammatory parameters, microbial abundance (phylum level) and metabolic pathways in A. C57BL/6 and B. BALB/c mice. (DOCX) [file pone.0264400.s012.docx]

**S8A Table. Correlation analysis between inflammatory parameters, microbial abundance (phylum level) and metabolic pathways in C57BL/6 mice.**

|  | 1 | 2 | 3 | 4 | 5 | 6 | 7 | 8 | 9 | 10 | 11 | 12 | 13 | 14 | 15 | 16 | 17 | 18 | 19 | 20 | 21 |
| --- | --- | --- | --- | --- | --- | --- | --- | --- | --- | --- | --- | --- | --- | --- | --- | --- | --- | --- | --- | --- | --- |
| 1 | 1.00 |  |  |  |  |  |  |  |  |  |  |  |  |  |  |  |  |  |  |  |  |
| 2 | 0.30 | 1.00 |  |  |  |  |  |  |  |  |  |  |  |  |  |  |  |  |  |  |  |
| 3 | 0.99 | 0.32 | 1.00 |  |  |  |  |  |  |  |  |  |  |  |  |  |  |  |  |  |  |
| 4 | 0.98 | 0.15 | 0.98 | 1.00 |  |  |  |  |  |  |  |  |  |  |  |  |  |  |  |  |  |
| 5 | 0.96 | 0.03 | 0.96 | 0.99 | 1.00 |  |  |  |  |  |  |  |  |  |  |  |  |  |  |  |  |
| 6 | 0.86 | 0.22 | 0.85 | 0.93 | 0.97 | 1.00 |  |  |  |  |  |  |  |  |  |  |  |  |  |  |  |
| 7 | 0.99 | 0.35 | 0.99 | 0.98 | 0.95 | 0.84 | 1.00 |  |  |  |  |  |  |  |  |  |  |  |  |  |  |
| 8 | 0.99 | 0.24 | 0.99 | 0.99 | 0.98 | 0.89 | 0.99 | 1.00 |  |  |  |  |  |  |  |  |  |  |  |  |  |
| 9 | 0.99 | 0.17 | 0.99 | 0.99 | 0.99 | 0.92 | 0.98 | 0.99 | 1.00 |  |  |  |  |  |  |  |  |  |  |  |  |
| 10 | -0.74 | -0.42 | -0.73 | -0.84 | -0.90 | -0.98 | -0.71 | -0.78 | -0.83 | 1.00 |  |  |  |  |  |  |  |  |  |  |  |
| 11 | 0.93 | 0.07 | 0.92 | 0.98 | 0.99 | 0.99 | 0.91 | 0.95 | 0.97 | -0.93 | 1.00 |  |  |  |  |  |  |  |  |  |  |
| 12 | -0.39 | -0.76 | -0.37 | -0.52 | -0.62 | -0.80 | -0.34 | -0.44 | -0.51 | 0.91 | -0.70 | 1.00 |  |  |  |  |  |  |  |  |  |
| 13 | 0.87 | 0.21 | 0.86 | 0.94 | 0.97 | 0.99 | 0.84 | 0.90 | 0.93 | -0.98 | 0.99 | -0.79 | 1.00 |  |  |  |  |  |  |  |  |
| 14 | 0.99 | 0.27 | 0.99 | 0.99 | 0.97 | 0.88 | 0.99 | 0.99 | 0.99 | -0.76 | 0.94 | -0.42 | 0.89 | 1.00 |  |  |  |  |  |  |  |
| 15 | -0.92 | -0.10 | -0.91 | -0.97 | -0.99 | -0.99 | -0.90 | -0.94 | -0.96 | 0.94 | -0.99 | 0.72 | -0.99 | -0.93 | 1.00 |  |  |  |  |  |  |
| 16 | -0.98 | -0.10 | -0.97 | -0.99 | -0.99 | -0.95 | -0.97 | -0.99 | -0.99 | 0.86 | -0.99 | 0.57 | -0.95 | -0.98 | 0.98 | 1.00 |  |  |  |  |  |
| 17 | -0.95 | -0.02 | -0.94 | -0.98 | -0.99 | -0.98 | -0.93 | -0.96 | -0.98 | 0.92 | -0.99 | 0.66 | -0.98 | -0.96 | 0.99 | 0.99 | 1.00 |  |  |  |  |
| 18 | 0.85 | 0.24 | 0.84 | 0.92 | 0.96 | 0.99 | 0.83 | 0.88 | 0.92 | -0.98 | 0.98 | -0.81 | 0.99 | 0.87 | -0.99 | -0.94 | -0.98 | 1.00 |  |  |  |
| 19 | -0.08 | -0.97 | -0.10 | -0.07 | -0.19 | -0.43 | -0.13 | -0.02 | -0.06 | 0.61 | -0.29 | 0.89 | -0.42 | -0.05 | 0.32 | 0.13 | 0.25 | -0.45 | 1.00 |  |  |
| 20 | -0.46 | -0.98 | -0.48 | -0.32 | -0.20 | -0.05 | -0.51 | -0.41 | -0.34 | 0.25 | -0.11 | 0.64 | -0.03 | -0.43 | 0.08 | 0.27 | 0.15 | -0.07 | 0.92 | 1.00 |  |
| 21 | 0.97 | 0.53 | 0.97 | 0.92 | 0.86 | 0.71 | 0.98 | 0.95 | 0.93 | -0.55 | 0.81 | -0.15 | 0.72 | 0.96 | -0.79 | -0.90 | -0.84 | 0.70 | -0.33 | -0.67 | 1.00 |

**S8B Table. Correlation analysis between inflammatory parameters, microbial abundance (phylum level) and metabolic pathways in BALB/c mice.**

|  | 1 | 2 | 3 | 4 | 5 | 6 | 7 | 8 | 9 | 10 | 11 | 12 | 13 | 14 | 15 | 16 | 17 | 18 | 19 | 20 | 21 |
| --- | --- | --- | --- | --- | --- | --- | --- | --- | --- | --- | --- | --- | --- | --- | --- | --- | --- | --- | --- | --- | --- |
| 1 | 1.00 |  |  |  |  |  |  |  |  |  |  |  |  |  |  |  |  |  |  |  |  |
| 2 | 0.10 | 1.00 |  |  |  |  |  |  |  |  |  |  |  |  |  |  |  |  |  |  |  |
| 3 | 0.74 | 0.60 | 1.00 |  |  |  |  |  |  |  |  |  |  |  |  |  |  |  |  |  |  |
| 4 | 0.80 | 0.52 | 0.99 | 1.00 |  |  |  |  |  |  |  |  |  |  |  |  |  |  |  |  |  |
| 5 | 0.01 | 1.00 | 0.67 | 0.59 | 1.00 |  |  |  |  |  |  |  |  |  |  |  |  |  |  |  |  |
| 6 | 0.22 | 0.95 | 0.82 | 0.76 | 0.97 | 1.00 |  |  |  |  |  |  |  |  |  |  |  |  |  |  |  |
| 7 | 0.05 | 0.99 | 0.71 | 0.64 | 0.99 | 0.98 | 1.00 |  |  |  |  |  |  |  |  |  |  |  |  |  |  |
| 8 | 0.49 | 0.82 | 0.95 | 0.91 | 0.87 | 0.96 | 0.90 | 1.00 |  |  |  |  |  |  |  |  |  |  |  |  |  |
| 9 | 0.18 | 1.00 | 0.53 | 0.45 | 0.99 | 0.92 | 0.97 | 0.77 | 1.00 |  |  |  |  |  |  |  |  |  |  |  |  |
| 10 | -0.72 | -0.62 | -0.99 | -0.99 | -0.69 | -0.84 | -0.73 | -0.96 | -0.56 | 1.00 |  |  |  |  |  |  |  |  |  |  |  |
| 11 | 0.05 | 1.00 | 0.64 | 0.56 | 0.99 | 0.96 | 0.99 | 0.85 | 0.99 | -0.66 | 1.00 |  |  |  |  |  |  |  |  |  |  |
| 12 | -0.34 | -0.97 | -0.39 | -0.29 | -0.94 | -0.84 | -0.92 | -0.66 | -0.99 | 0.42 | -0.96 | 1.00 |  |  |  |  |  |  |  |  |  |
| 13 | 0.15 | 0.99 | 0.55 | 0.47 | 0.99 | 0.93 | 0.98 | 0.79 | 0.99 | -0.58 | 0.99 | -0.98 | 1.00 |  |  |  |  |  |  |  |  |
| 14 | 0.12 | 0.99 | 0.58 | 0.50 | 0.99 | 0.94 | 0.99 | 0.81 | 0.99 | -0.61 | 0.99 | -0.97 | 0.99 | 1.00 |  |  |  |  |  |  |  |
| 15 | -0.48 | -0.92 | -0.23 | -0.14 | -0.88 | -0.74 | -0.85 | -0.53 | -0.95 | 0.26 | -0.90 | 0.99 | -0.94 | -0.93 | 1.00 |  |  |  |  |  |  |
| 16 | -0.95 | -0.41 | -0.49 | -0.57 | -0.32 | -0.10 | -0.27 | -0.19 | -0.48 | 0.46 | -0.36 | 0.62 | -0.46 | -0.42 | 0.74 | 1.00 |  |  |  |  |  |
| 17 | 0.53 | -0.89 | -0.17 | -0.08 | -0.85 | -0.70 | -0.82 | -0.48 | -0.93 | 0.21 | -0.87 | 0.98 | -0.92 | -0.90 | 0.99 | 0.77 | 1.00 |  |  |  |  |
| 18 | 0.06 | 0.99 | 0.72 | 0.65 | 0.99 | 0.99 | 0.99 | 0.90 | 0.97 | -0.74 | 0.99 | -0.92 | 0.98 | 0.98 | -0.84 | -0.26 | -0.81 | 1.00 |  |  |  |
| 19 | -0.79 | -0.53 | -0.99 | -0.99 | -0.61 | -0.78 | -0.66 | -0.92 | -0.47 | 0.99 | -0.58 | 0.32 | -0.49 | -0.52 | 0.16 | 0.55 | 0.10 | -0.66 | 1.00 |  |  |
| 20 | -0.24 | -0.94 | -0.83 | -0.78 | -0.97 | -0.99 | -0.98 | -0.97 | -0.91 | 0.85 | -0.96 | 0.83 | -0.92 | -0.93 | 0.73 | 0.07 | 0.69 | -0.98 | 0.79 | 1.00 |  |
| 21 | 0.27 | 0.98 | 0.45 | 0.36 | 0.96 | 0.88 | 0.95 | 0.71 | 0.99 | -0.48 | 0.97 | -0.99 | 0.99 | 0.99 | -0.97 | -0.56 | -0.96 | 0.94 | -0.38 | -0.87 | 1.00 |

**Abbreviations:** 1,*TLR2*; 2, *TLR4*; 3, *TNFα*; 4, *IFNγ*; 5, *IL1b*; 6, *IL6*; 7, *IL12*; 8, *IL17*; 9, *IL21*; 10, *IL10*; 11, *Cldn2*; 12, *ZO1*; 13, Gut Permeability; 14, C-Reactive Protein; 15, Bacteroidetes; 16, Verrucomicrobia; 17, Firmicutes; 18, Proteobacteria; 19, Carbohydrate Metabolism; 20, Nucleotide Metabolism; 21, Amino acid and Lipid Metabolism
