# Supplementary material for: Differential colitis susceptibility of Th1- and Th2-biased mice: A multi-omics approach
Source: PLoS One. 2022 Mar 9;17(3):e0264400. doi: 10.1371/journal.pone.0264400 (PMC8906622; doi:10.1371/journal.pone.0264400)
Supplement: Attachment — Submitted filename: Response2Reviewers.docx [file pone.0264400.s013.docx]

**School of Biological Sciences**

NATIONAL INSTITUTE OF SCIENCE EDUCATION AND RESEARCH

(NISER, An autonomous Institute under Dept. of Atomic Energy, Govt. of India)

---------------------------------------------------------------------------------------------------------------------

Wednesday, 24 November 2021

Dr. Palok Aich, Associate Professor, Ramalingaswami Fellow.

Hon. Diploma: Medical University, Varna, Bulgaria

Formerly:

- 1^st^ Dean, International Affairs & Resource Planning
- 1^st^ Chairperson,
  - School of Biological Sciences
  - Under Graduate Committee of the Institute
- Faculty-in-charge (FIC)
  - Library,
  - Institute Works Department

URL:  <https://www.niser.ac.in/users/palokaich>

Professor Lili Chen,

Academic Editor,

PLoS One

Dear Professor Chen,

Thank you for your email of Nov 1 regarding

PONE-D-21-31676

Differential colitis susceptibility of Th1- and Th2-biased mice- A Multi-Omics Approach

PLOS ONE

We thank you and the reviewers for your valuable suggestions and critical comments to improve the manuscript quality. We have now addressed all the queries and responded in line in red below each comment.

**General comments**

When submitting your revision, we need you to address these additional requirements.

1. Please ensure that your manuscript meets PLOS ONE's style requirements, including those for file naming. The PLOS ONE style templates can be found at 
<https://journals.plos.org/plosone/s/file?id=wjVg/PLOSOne_formatting_sample_main_body.pdf> and 
<https://journals.plos.org/plosone/s/file?id=ba62/PLOSOne_formatting_sample_title_authors_affiliations.pdf>.

Answer: We have edited as per PLOS ONE style guidelines.

2. To comply with PLOS ONE submissions requirements, in your Methods section, please provide additional information regarding the experiments involving animals and ensure you have included details on (1) methods of sacrifice, (2) methods of anesthesia and/or analgesia, and (3) efforts to alleviate suffering.

Answer: We have mentioned the methods of sacrifice and the measures taken to alleviate the suffering of the animals during experimental procedures in between line no. 126-131.

3. Thank you for stating the following financial disclosure: 
 [The funders had no role in study design, data collection and analysis, decision to publish, or preparation of the manuscript.]

At this time, please address the following queries:

a) Please clarify the sources of funding (financial or material support) for your study. List the grants or organizations that supported your study, including funding received from your institution. 
b) State what role the funders took in the study. If the funders had no role in your study, please state: “The funders had no role in study design, data collection and analysis, decision to publish, or preparation of the manuscript.”
c) If any authors received a salary from any of your funders, please state which authors and which funders.
d) If you did not receive any funding for this study, please state: “The authors received no specific funding for this work.”

Please include your amended statements within your cover letter; we will change the online submission form on your behalf.

Answer: The funding and financial resources used in the current study are mentioned in the funding section (Line No. 1410-1413).

4. We note that you have stated that you will provide repository information for your data at acceptance. Should your manuscript be accepted for publication, we will hold it until you provide the relevant accession numbers or DOIs necessary to access your data. If you wish to make changes to your Data Availability statement, please describe these changes in your cover letter and we will update your Data Availability statement to reflect the information you provide.

Answer: We will provide the public accession of all high-throughput data upon formal acceptance of the current manuscript in the journal.

5. We note that you have included the phrase “data not shown” in your manuscript. Unfortunately, this does not meet our data sharing requirements. PLOS does not permit references to inaccessible data. We require that authors provide all relevant data within the paper, Supporting Information files, or in an acceptable, public repository. Please add a citation to support this phrase or upload the data that corresponds with these findings to a stable repository (such as Figshare or Dryad) and provide and URLs, DOIs, or accession numbers that may be used to access these data. Or, if the data are not a core part of the research being presented in your study, we ask that you remove the phrase that refers to these data.

Answer: We have rephrased the statement “ data not shown” and provided the data as disease severity/ clinical score in the S1A figure (Line No. 386-387).

6.  Please review your reference list to ensure that it is complete and correct. If you have cited papers that have been retracted, please include the rationale for doing so in the manuscript text, or remove these references and replace them with relevant current references. Any changes to the reference list should be mentioned in the rebuttal letter that accompanies your revised manuscript. If you need to cite a retracted article, indicate the article’s retracted status in the References list and also include a citation and full reference for the retraction notice.
Answer: We have updated all the references as per mentioned guidelines.


Reviewers' comments:

Reviewer's Responses to Questions

Comments to the Author

1. Is the manuscript technically sound, and do the data support the conclusions?

The manuscript must describe a technically sound piece of scientific research with data that supports the conclusions. Experiments must have been conducted rigorously, with appropriate controls, replication, and sample sizes. The conclusions must be drawn appropriately based on the data presented.

Reviewer #1: Yes

Reviewer #2: Yes

2. Has the statistical analysis been performed appropriately and rigorously?

Reviewer #1: Yes

Reviewer #2: Yes

3. Have the authors made all data underlying the findings in their manuscript fully available?

The [PLOS Data policy](http://www.plosone.org/static/policies.action#sharing) requires authors to make all data underlying the findings described in their manuscript fully available without restriction, with rare exception (please refer to the Data Availability Statement in the manuscript PDF file). The data should be provided as part of the manuscript or its supporting information, or deposited to a public repository. For example, in addition to summary statistics, the data points behind means, medians and variance measures should be available. If there are restrictions on publicly sharing data—e.g. participant privacy or use of data from a third party—those must be specified.

Reviewer #1: Yes

Reviewer #2: Yes

4. Is the manuscript presented in an intelligible fashion and written in standard English?

PLOS ONE does not copyedit accepted manuscripts, so the language in submitted articles must be clear, correct, and unambiguous. Any typographical or grammatical errors should be corrected at revision, so please note any specific errors here.

Reviewer #1: Yes

Reviewer #2: Yes

5. Review Comments to the Author

Please use the space provided to explain your answers to the questions above. You may also include additional comments for the author, including concerns about dual publication, research ethics, or publication ethics. (Please upload your review as an attachment if it exceeds 20,000 characters)

Reviewer #1: Mukhopadhyay et al performed experiments and analyses aimed at addressing the potential mechanisms underlying the differential colitis susceptibility between C57BL/6 (type 1-prone) and BALB/c (type 2-prone) mice. They employed multi-omics approaches including comparative transcriptomics, metabonomics, and metagenomics analyses. They found that, in C57BL/6, the severity of DSS-induced colitis was mainly host factor-driven, including pro-inflammatory host genes and host metabolites. On the contrary, in BALB/c, the disease severity was more affected by the microbiota, such as gut microbial composition and microbial metabolites. These are very useful datasets which could be very helpful for other investigators in choosing mouse strains to study DSS-colitis or other disease models, as well as in considering the candidate pro-/anti-inflammatory host genes, host metabolites, microbial strains, or microbial metabolites reported in this manuscript as potential mechanisms.

I only have some minor comments:

1. Lines 210 to 213 - “The shortlisted genes from the transcriptomics data are mainly related to the inflammatory responses of the host. In C57BL/6, genes were primarily associated with pro-inflammation, whereas in BALB/c, genes were related to anti-inflammation.” Given that these conclusion are foundations of this manuscript, I would suggest the authors to use a heat map to show the shortlisted genes as a part of a main figure.

Answer: We appreciate the efforts of the reviewers. We tried to incorporate the valuable comments by the reviewers. As reviewer #1 suggested to use a heatmap of shortlisted genes as the main figure for a better understanding of the data. We have followed the instructions and incorporated the heatmaps in the main figure (Figure 3P and 3Q ) and also explained in detail in the main text of the manuscript (Line No. 603-605) and also in figure legends (Line no. 638-640).

2. Tables S3, S4 and S5 were only mentioned in the Materials and Methods section which is a waste of good data. I would suggest the authors to cite these tables in their main text.

Answer: We have incorporated and cited all the three tables in the main text as per the reviewer’s suggestion. Table S3 (Line No. 605), Table S4 (Line No. 644), Table S5 (Line No. 645) all three tables were well explained in the main text.

Reviewer #2: The manuscritps presents a thorough analysis of DSS induced colitis in Th1 and Th2 biased mice strains, C57BL/6 and BALB/c resectively. In order to find clinical differences between strains, they use first a high dose of DSS (1 week), followed by a lower dose (1 week). En the second week of treatment BALB/c mice recover, while C57BL/6 don´t. Surprisingly, this results are not shown, although they could go to a suplementary figure. No data on clinical scores, hystology or bowel weight/length is presented, making results weaker. A simple figure describing the findings in this particular model should be included.

Answer: We thank the reviewer #2 for this comment. We have incorporated the data of clinical and histopathological scores and bowel length of the animal at control and DSS treated conditions. We have provided the data as supplementary figure (S1_Fig) and also mentioned in the main text in the appropriate places (Line No. 385-392). As reviewer suggested to provide a simple figure describing the model in a better way. We have provided the major and unique findings of the current model as supplementary figure (S2_Fig) and mentioned in the manuscript's main text (Line No. 393-394).

Than the authors study cytokines (Pro-inflamatory and tolerogenic) at different stages. Results regarding cytokines are quite consisting with other papers. The multiomics approach, regarding bowel genes and metabolites compares to changes in gut microbiota are a unique and highly interesting contribution of the article. The data analysis are sound and rigorous statiscal test are performed. The English is quite clear and easy to read.
The results are well discussed and enrich the article.
